# Primary Breast Adenocarcinoma in Ectopic Breast Tissue in the Vulva

**DOI:** 10.1155/2013/721696

**Published:** 2013-08-26

**Authors:** Jason McMaster, Anahita Dua, Sean C. Dowdy

**Affiliations:** ^1^Department of Obstetrics and Gynecology, Division of Gynecologic Surgery, Mayo Clinic, Rochester, MN 55905, USA; ^2^Department of Obstetrics and Gynecology, Medical College of Wisconsin, Milwaukee, WI 53226, USA; ^3^Department of Surgery, Medical College of Wisconsin, Milwaukee, WI 53226, USA; ^4^Department of Surgery, Center for Translational Injury Research (CeTIR), University of Texas-Houston, MSB 5.030, 6413 Fannin Street, Houston, TX 77030, USA

## Abstract

*Introduction*. Accessory breast tissue is a rare finding in the general population with an incidence of 1-2%. An even rarer occurrence is accessory breast tissue afflicted with breast carcinoma. We present a brief report discussing diagnosis and management of a patient who presented with primary breast adenocarcinoma in vulval supranumerary tissue. *Brief Report*. A 60-year-old Caucasian female presented with a lesion in her left vulva that she first identified during adolescence. The lesion began to grow and ulcerate prompting her to receive treatment. Biopsy was inconclusive, and metastatic workup was negative, so her lesion was treated as an isolated breast lump and removed via wide local excision. *Conclusion*. Primary breast adenocarcinoma of the vulva is exceedingly rare. A paucity of the literature on this topic unfortunately means that strong evidence does not exist detailing the best management of this patient cohort. However, given that histological data confirms these cancers are virtually the same as breast cancers, it logically follows that the best treatment practices for breast cancer may be applied to treat these patients presenting with primary vulva cancers of ectopic breast tissue.

## 1. Introduction

Accessory breast tissue is a rare finding in the general population with an incidence of  1-2% [1]. An even rarer occurrence is accessory breast tissue afflicted with breast carcinoma [1–3]. Embryologically, bilateral mammary ectodermal ridges extend ventrally from the axillary folds to the inguinal region. Ectopic breast tissue is most commonly reported in the axilla but can be present at any point within this primitive embryological milk line. Ectopic breast tissue may contain all breast constituents; areola: nipple, or parenchyma [1]. This tissue responds to physiological stresses and hormonal stimulation in a similar way as normally located breast tissue; as a result it can present with benign or malignant breast pathology. The specific occurrence of vulvar primary adenocarcinoma of ectopic mammary tissue is exceptionally rare with approximately 25 cases being reported in the English literature between 1872 and 2012 [3]. 

We present a brief report discussing diagnosis and management of a patient who presented with primary breast adenocarcinoma in vulval supranumerary tissue. 

## 2. Case Report 

A 60-year-old G0P0 Caucasian female presented with a lesion in her left vulva that she first identified during adolescence. She reported overall good health with no significant medical or surgical history and had undergone regular cervical cancer screening and breast cancer screening which has always been normal. She had a family history positive for breast cancer in her mother. Six months prior to presentation, the vulvar lesion rapidly increased in size with the beginnings of ulceration. She incurred minimal bleeding and discomfort from this and did not seek immediate medical attention. The lesion continued to enlarge which prompted her to consult a physician. 

On inspection of her vulva, a 3 cm pedunculated, round, and ulcerated mass was identified on the left labium majus. The surrounding skin appeared normal, and no other vulva pathology was identified. An inguinal lymph node survey was negative for masses. Speculum exam of the cervix revealed no abnormalities. 

A biopsy of the lesion was inconclusive. CT scan of the abdomen and pelvis with IV contrast showed no metastasis, no adenopathy, and no soft tissue thickening at the vulva. A colonoscopy and CA-125 levels were performed which revealed no abnormalities. At this point we decided to proceed with local excision given the negative metastatic workup and the shallow base of the lesion. We performed a wide local excision of the left vulva with 1 cm margins. Frozen pathology revealed negative margins and no angiolymphatic invasion. We elected to omit inguinal lymphadenectomy given the pedunculated nature of the lesion with a narrow stalk. 

Final pathology reported a grade 4 invasive adenocarcinoma forming a 1.9 × 1.8 × 1.3 cm mass with a depth of 0.5 cm. Margins were negative for cancer. Immunoperoxidase studies were performed on paraffin sections of the specimen using antibodies directed against the following antigens: CK7, ER, GCDFP-15, and mammaglobin. The tumor cells were strongly positive for CK7, ER, and mammaglobin. The morphology of the tumor cells and immunoprofile were suggestive of breast carcinoma. This was a very unusual case of vulvar adenocarcinoma without involvement of any glandular structures or any associated Paget's disease. 

The patient was discharged home the day following surgery. Her discharge diagnosis was adenocarcinoma in ectopic breast tissue of the left vulva. The final pathologic review was interesting as this was pure adenocarcinoma arising from ectopic breast tissue. The tumor was positive for estrogen receptors and mammaglobin. 

Radiation oncology was consulted to determine if the patient would benefit from adjuvant treatment. They agreed that her lesion be treated as a lumpectomy for breast cancer. At one month followup, the wound was well healed and no other lesions were present. The patient resides in another state and was agreeable to pursuing radiation therapy closer to her home. 

## 3. Discussion 

The first reported case of ectopic breast tissue of the vulva was reported by Hartung in 1872 when he described a fully formed breast in the left labia majora [4]. The cases that followed this initial description were of varying degrees of anatomical development and functionality of which only a few have been histologically confirmed cases of primary breast adenocarcinoma of the vulva. This is an exceedingly rare carcinoma presentation and only a handful of case studies exist as evidence on which to base clinical management. To date 25 cases have been reported involving vulva infiltrating carcinoma [3]. As was the case with our patient, this cohort classically presents with a history of an innocuous lesion in the vulva area that was noted in their premenopausal years [1]. The lesion has no typical characteristics and does not cause pain, so it may go ignored for years. The only way to determine pathological significance of the lesion is via biopsy with required immunohistochemistry to determine the tumor type and prognosis. For diagnostics purposes, antibodies associated with breast carcinoma include estrogen receptor (ER), progesterone receptor (PR), gross cystic fluid protein (GCDFP), HER-2/neu, cytokeratins (CK5/6, CK7, CK20), and the mucin glycoprotein antibodies, namely, MUC2, MUC3, MUC5AC, MUC6, and DAS-1. Of this group, estrogen, progesterone, and GCDFP are exceptionally specific for breast carcinoma. The literature would suggest that these tumors, aside from their unique location, act strictly as an equivalent breast mass would in regards to hormonal responsiveness and aggressiveness [1–4]. Therefore, appropriate management of such lesions mirrors the evidence-based management protocols in place for breast cancer. 

The majority of case reports in the literature detail management involving a wide local excision or radical vulvectomy with node dissection followed by adjuvant chemotherapy, hormonal therapy, or radiation, echoing the management of an isolated malignant breast mass. Further tailored therapy depends on histological tumor type as was the case with our patient. At present, patients with ER positive vulva cancer have been offered Tamoxifen as an adjuvant hormonal therapy given that it has been proven to increase survival in patients with ER positive breast malignancies. One study from the UK discussed the use of Anastrozole on an 81-year-old female with primary lobular breast cancer of the vulva given that it is at least as effective as Tamoxifen in postmenopausal women [5]. Unfortunately, this case report did not give further details as to the patient's outcome, and a case report would not suffice as appropriate evidence to adopt this practice in place of Tamoxifen. 

In 2006, Abbott and Ahmed [6] of Mayo Clinic Department of Dermatology reviewed 19 cases reported in the literature and described a 51-year-old patient with a long-standing nodule on her right interlabial sulcus. Excisional biopsy and Mohs micrographic surgery demonstrated an infiltrating adenocarcinoma of the mammary-like glands involving the dermis [6]. 

This review of 20 cases found that the mean age at diagnosis was 59.6 years, the labia majora were involved in 13 cases (65%), and the mean lesional size was 2.5 cm [6]. In keeping with our literature search, they reported that tumor histologic patterns varied significantly and the criteria for establishing the diagnosis of adenocarcinoma of mammary-like glands included identifying transition zones between normal mammary-like glands and adenocarcinomatous areas. Abbott and Ahmed [6] concluded that aggressive surgical therapeutic regimens, particularly in the case of tumors localized to the skin, should be reassessed, given the morbidity faced by such therapy. Rather, they argued for Mohs micrographic surgery suggesting that alternate management may be adopted for tumors localized to the skin, especially in elderly patients [6]. This tumor localization to the skin is unique to ectopic breast carcinoma given that cancer of the breast itself is not characterized as such if localized to skin only. 

## 4. Conclusion

Primary breast adenocarcinoma of the vulva is exceedingly rare. A paucity of the literature on this topic unfortunately means that strong evidence does not exist detailing the best management of this patient cohort. However, given that histological data confirms that these cancers are virtually the same as breast cancers, it logically follows that the best treatment practices for breast cancer may be applied to treat these patients presenting with primary vulva cancers of ectopic breast tissue. 
